# The microwave-assisted synthesis of silica nanoparticles and their applications in a soy plant culture†

**DOI:** 10.1039/d3ra05648a

**Published:** 2023-09-18

**Authors:** Daniel Carneiro Freitas, Italo Odone Mazali, Fernando Aparecido Sigoli, Danielle da Silva Francischini, Marco Aurélio Zezzi Arruda

**Affiliations:** a Spectrometry, Sample Preparation and Mechanization Group, Institute of Chemistry, University of Campinas – Unicamp P.O. Box 6154 Campinas SP 13083-970 Brazil zezzi@unicamp.br; b National Institute of Science and Technology for Bioanalytics, Institute of Chemistry, University of Campinas – Unicamp P.O. Box 6154 Campinas SP 13083-970 Brazil; c Functional Materials Laboratory – Institute of Chemistry, University of Campinas – UNICAMP P. O. Box 6154 13083-970 Campinas SP Brazil

## Abstract

A rapid and environmentally friendly synthesis of thermodynamically stable silica nanoparticles (SiO_2_-NPs) from heating *via* microwave irradiation (MW) compared to conductive heating is presented, as well as their evaluations in a soy plant culture. The parameters of time and microwave power were evaluated for the optimization of the heating program. Characterization of the produced nanomaterials was obtained from the dynamic light scattering (DLS) and zeta potential analyses, and the morphology of the SiO_2_-NPs was obtained by transmission electron microcopy (TEM) images. From the proposed synthesis, stable, monodisperse, and amorphous SiO_2_-NPs were obtained. Average sizes reported by DLS and TEM techniques were equal to 11.6 nm and 13.8 nm, respectively. The water-stable suspension of SiO_2_-NPs shows a zeta potential of −31.80 mV, and the homogeneously spheroidal morphology observed by TEM corroborates with the low polydispersity values (0.300). Additionally, the TEM with fast Fourier transform (FFT), demonstrates the amorphous characteristic of the nanoparticles. The MW-based synthesis is 30 times faster, utilizes 4-fold less reagents, and is *ca.* 18-fold cheaper than conventional synthesis through conductive heating. After the synthesis, the SiO_2_-NPs were added to the soil used for the cultivation of soybeans, and the homeostasis for Cu, Ni, and Zn was evaluated through the determination of their total contents by inductively coupled plasma mass spectrometry (ICP-MS) in soy leaves and also through bioimages obtained using laser ablation inductively coupled plasma mass spectrometry (LA-ICP-MS). Although the results corroborate through both techniques, they also show the influence of these nanoparticles on the elemental distribution of the leaf surface with altered homeostasis of such elements from both transgenic crops compared to the control group.

## Abstract 2

A rapid and environmentally friendly synthesis of thermodynamically stable silica nanoparticles (SiO_2_-NPs) from heating *via* microwave irradiation (MW) compared to conductive heating is presented, as well as their evaluations in a soy plant culture.

## Graphical abstract

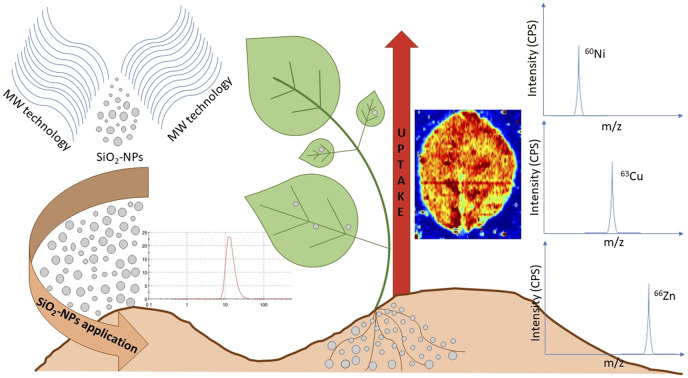

## 1. Introduction

Nanotechnology is one of the most rapidly developing areas of science,^1,2^ which can be explained by the global industrial revolution of the 21^st^ century.^3–6^ At present, this is a leading technology within a diversity of research areas and interdisciplinary fields^7–14^ enabling testing, controlling, regulating, modifying, processing, producing, and using structures in which at least one of the dimensions does not exceed 100 nanometers.^3,15,16^

The use of nanoparticles (NPs) in agriculture has been widely employed in order to obtain healthy and safe food. Thus, the applications of these materials in studies of this nature have been explored as alternatives to agricultural inputs and in the absorption and translocation of micro- and macronutrients from the soil to the plants.^9^ Agriculture, food, and natural resources contribute to challenges like sustainability, susceptibility, and human health, and the application of nanomaterials in agriculture objectives reduces the amount of applied chemicals, minimizes nutrient losses in fertilization, and increases yield through pest and nutrient management.^12^

Nanotechnology has the prospective to improve the agriculture and food industry with novel nanotools for controlling rapid disease spread and enhancing the capacity of plants to absorb nutrients, among others.^9,12^

There are various forms of remediation in plants, such as biomass, microorganism, and also NPs in protecting the plant from growth to obtaining adult plants.^17–21^

In this sense, in order to obtain nanoparticles some new strategies can be pointed out, such as green synthesis, and the use of alternative energies such as microwaves. In the first case, it presents less aggressive conditions to the environment, since a diversity of microorganisms, low biomolecules, polysaccharides, and biomass can be used as stabilizers as the main route of the synthesis. In the second case, the irradiation through microwave technology has proven beneficial since the more uniform heating, through the phenomena of rotation of dipoles and ionic migration, the nucleation and growth processes allow slight changes in reaction temperature and/or time reproducibly result in structures with the desired properties since the process can be better controlled to rapidly obtain thermodynamically stable and monodisperse nanoparticles in terms of size and morphology.^3,22^ In most cases, microwave-mediated reaction times are substantially shorter, and particle size distribution is much smaller than compared to conventional methods.

Then, allying all the facilities to work with microwave technology, an due to the importance of silica nanoparticles (SiO_2_-NPs) in the agriculture, studies with silica and silica nanoparticles are employed in various ways, including the mitigation of various crops to biotic and abiotic factors,^23^ as mitigating agent for potentially toxic metals,^24^ flavor carriers, marking fruits and vegetables, and anticaking agents, among others.^9^ In fact, silica nanoparticles are nanomaterials formed from the nucleation and growth processes of silicate ions when subjected to heating, they are mainly classified into solid, non-porous, and mesoporous.^25^

In this sense, fast and efficient synthesis methods, like microwave irradiation, show promise to produce more suitable SiO_2_-NPs with desirable characteristics in terms of size distribution, morphology, and stability, which directly influence their physical, chemical, electrical, and catalytic properties and their toxicity.^26–28^ Their structural properties include large surface areas and pore diameters from 2 up to 50 nm, being produced using aqueous synthesis methods based on conductive heating, during which the aggregation or possible degradation of SiO_2_-NPs is undesirable and requires considerable attention.

Recent studies report the influence of the size and stability of SiO_2_-NPs in a variety of applications, numerous innovative nanomaterials have been developed to improve food quality and safety, crop growth and monitoring of environmental conditions, and also as nanopesticides and nanofertilizers.^9,29–31^

In this way, we are proposing a route for faster and cheaper synthesis of SiO_2_-NPs using MW technology and environmentally friendly reagents once our proposal use no toxic reagent and no organic stabilizer for obtaining the silica nanoparticles. After synthesis, the NPs were then applied to the soybean cultivation for qualitative evaluation, through bioimages using LA-ICP-MS, and quantitative evaluation, through ICP-MS, of Cu, Ni, and Zn homeostasis during the development of this culture with low water a day.

## 2. Materials and methods

### 2.1 SiO_2_-NP synthesis

First of all, the real concentration of the SiO_2_·NaOH stock solution utilized for SiO_2_-NPs synthesis was determined by gravimetric method^32^ using sodium silicate solution (Sigma-Aldrich 37.28% m/v). Two synthesis routes were used for this work, namely: (1) conductive heating (with the use of a reflux system) and (2) microwave heating. To provide the synthesis of SiO_2_-NPs and as a way of comparing the results with each other, two different strategies were adopted in order to obtain silica-based nanomaterials with spherical shape and homogeneous size distribution.

Thus, for the synthesis using conductive heating,^32^ two different solutions were used. The first had a concentration of 1% m/v of sodium silicate from a stock solution in a total volume of 500 mL of water (18.2 MΩ cm; 25 °C), while the second had a concentration of 1 mol L^−1^ of citric acid (Sigma-Aldrich 99.5%) in a total volume of 500 mL of water. After the addition of sodium silicate, citric acid was gradually added until reaching a pH of 10.50 (final pH value for carrying out the synthesis). Then, it was refluxed in a silicone bath system with magnetic stirring for 24 h at constant temperature (90 °C). After the reaction, gradual cooling to room temperature was carried out.

For the synthesis of SiO_2_-NPs through microwave heating, a microwave oven (DGT 100 Plus, Provecto Analitica, Brazil) was used, following the same conditions considered for the conductive heating; however, 10.00 mL were used in each PTFE tube. Table 1 shows four different programs utilized for this task, in which time and power were varied for attaining the best conditions for SiO_2_-NP synthesis.

**Table 1:** Optimization of the silica nanoparticles synthesis method using different microwave heating program

Program	Power and time				
	1 step	2 step	3 step	4 step	5 step
1	0–400 W @ 5 min	400–700 W @ 15 min	700–400 W @ 10 min	400–0 W @ 20 min	—
2	0–400 W @ 5 min	400–700 W @ 15 min	700–400 W @ 10 min	400–200 W @ 10 min	200–0 W @ 10 min
3	0–400 W @ 5 min	400–700 W @ 15 min	700–400 W @ 10 min	400–200 W @ 5 min	200–0 W @ 15 min
4	0–400 W @ 5 min	400–700 W @ 15 min	700–400 W @ 5 min	400–200 W @ 5 min	200–0 W @ 20 min

To obtain a homogeneous heating system, twelve replicates of solutions were homogeneously distributed into the microwave oven for each heating program. At the end of each program, the resulting solutions were transferred to a polyethylene container and cooled in the refrigerator (at *ca.* 4 °C) until the characterization of the nanoparticles.

### 2.2 SiO_2_-NP characterization

In order to evaluate the performance of the syntheses, current techniques for nanoparticle characterization were used,^33^ such as DLS, the *ξ*-potential (using a Zetasizer Nano ZS, Malvern Instruments Ltd). Nanoparticle morphology was investigated in a Carl Zeiss Libra 120 Plus transmission electron microscope equipped with an in-column OMEGA filter, using a tungsten thermionic source at an acceleration voltage of 80 kV. Images were recorded with a Cantega (Olympus) 2k × 2k CCD camera. Sample was prepared by ultrasonicating for 20 min the nanoparticle aqueous dispersion and dripping 5 μL on the dull side of an ultrathin carbon coated copper grid (TedPella#1824). The suspension was left to settle for 3 min and dried using filter paper. The fast Fourier transform (FFT) of the images was performed using ImageJ® software to evaluate sample crystallinity. SiO_2_-NP suspensions in water (*ca.* 25 mg L^−1^) were sonicated for 10 minutes before preparing the grid, where 5 μL were used on the grid then dried completely in a desiccator.

For evaluating the total Si quantification, an inductively coupled plasma optical emission spectrometry (ICP OES) (Thermo Scientific, iCAP 6000 SERIES) operated in the axial position was used with a radio frequency (RF) of 1250 W and a gas flow of 0.4 L min^−1^ at 251.611 nm. To check the accuracy for the Si quantification, the Certified Reference Material – CRM 1640a (trace elements in natural water) was used.

### 2.3 SiO_2_-NP application to soy culture after synthesis

The soybean seeds employed in our study were kindly donated by Brejeiro Food Products (Orlândia, Brazil). Three soybean seeds were sown per plastic pot, with each pot containing *ca.* 50 g of a mixture of 1 : 1 (v/v) substrate (Tropstrato HT Hortaliças, Vida Verde™) and expanded vermiculite (Terra Master™). The plants were irrigated with enough deionized water to maintain the development of the plants for 6 days. Cultures were grown for 16 days in a growth chamber under a controlled temperature (27 °C ± 0.1 °C) and photoperiod (12 h). After 6 days of cultivation, the plants were grown under two irrigation conditions: control (water only) and SiO_2_-NPs at 400 mg kg^−1^. The applications of SiO_2_-NPs were made in mixtures of soil and vermiculite in equal volume proportions. The mass of the mixture (soil and vermiculite) was 50 g and the final concentration of SiO_2_-NPs was 400 mg kg^−1^. Each plant treated with SiO_2_-NPs had received the same amount of water as the control group at the end of the cultivation period. For this work, similar conditions were adopted as observed in the literature^34^ with some modifications.

The determination of micronutrients and other metals distributed on the leaves of transgenic RR and Intacta type soybean plants, grown in soils contaminated with SiO_2_-NPs at 400 mg kg^−1^, were performed according to the literature.^24^

### 2.4 Determination of Ni, Cu, and Zn in soy leaves by ICP-MS

For total Cu, Ni, and Zn quantification in leaves, analysis was performed *via* ICP-MS using a Shimadzu ICP-MS 2030 (Kyoto, Japan), and the following main parameters were utilized: RF 1200 W, plasma gas 9.0 L min-1, auxiliary gas 1.10 L min^−1^, carrier gas 0.7 L min^−1^, cell gas 6.0 L min^−1^, cell voltage −21 V, and energy filter 7.0 V. To check the accuracy for Cu, Ni, and Zn quantification, two Standard Reference Material – SRM 1573a (tomato leaves) and 1568a (rice flour), were used.

### 2.5 Bioimaging of Cu, Ni, and Zn in soy leaves through LA-ICP-MS

The bioimaging of the soy leaves at different conditions of cultivation was obtained for Cu, Ni, and Zn using the parameters presented in Table 2.

**Table 2:** LA-ICP-MS operating conditions for element determination

**ICP-MS conditions (PerkinElmer ELAN DRC-e)**	
RF power (W)	1000
Nebulizer gas flow (L min^−1^)	1.1
Auxiliary gas flow (L min^−1^)	1.8

**Data acquisition parameters**	
Reading mode	Peak hopping
Detector mode	Dual
Sweeps	3
Dwell time (ms)	20
Integration time (ms)	60
Monitored isotopes	^13^C(IS) ^66^Zn, ^60^Ni, ^63^Cu

**Laser conditions (New Wave, UP-213, USA)**	
Wavelength of Nd:YAG laser (nm)	213
Laser ablation intensity (%)	55
Frequency (Hz)	20
Spot size (μm)	80
Scan speed (μm s^−1^)	60
Average energy output (mJ)	0.257
Average fluence (J cm^−2^)	5.22
Warm up time (s)	7
Wash out time (s)	10

The distribution of metals was determined from the comparison of the contaminated crops with the control crop between the two transgenes mentioned above.^35^ In this case, leaf samples from each species were collected, and the elemental qualitative images were obtained by laser ablation inductively coupled plasma mass spectrometry (LA-ICP-MS) analysis.^34^ An open-source LA-iMageS software^36^ was used to build the final images, and the ^13^C isotope was applied as internal standard (IS) to correct instrument fluctuation.

## 3. Results and discussion

### 3.1 SiO_2_-NP synthesis

The concentration of SiO_2_·NaOH found in the precursor silicate solution was 37.28 ± 0.52% (m m^−1^). After suitable dilution for determination of Si *via* ICP OES, the concentration was 1.05 ± 0.02% (m/v), indicating a recovery of 100.15% (±0.10%).

The SiO_2_-NPs were synthesized using citric acid as a pH regulator and sodium silicate as a silicate ion source. In this study, microwave irradiation and suitable temperature were factors of great importance for the rapid and successful synthesis of SiO_2_-NPs. By changing the microwave irradiation conditions, particles of different sizes were able to be produce. The formation of SiO_2_-NPs was roughly detected by visual inspection of the Tyndall effect (see Fig. S1 – ESI†). No laser interaction was observed in the control sample, *i.e.* without the addition of SiO_2_ precursor. Thus, silica ions can be regrouped to the seed particles, with subsequent stabilization of these biomolecules present, from the citric acid in solution, for minimal pH variation during this process. Microwave irradiation enables rapid nucleation and growth of the particles, similar such as silver NPs found by Shu *et al.*, (2020),^37^ Yatao Wang *et al.*, (2023)^26^ and Magdalena Borowska *et al.*, (2023).^27^

From this visual parameter, it became possible to proceed with the other characterizations. Thus, four methods presented in Table 1 were developed and characterized. The method 4 presented greater intensity of the Tyndall effect while observing a formation of NPs of greater size and greater stability. While in method 1, smaller sizes of the materials and shorter exposure time to MW radiation were observed, for this same reason that methods 2 and 3 were not performed morphology characterizations. However, other non-invasive characterizations were performed for the methods presented in Table 1 and it was observed that from method 2, nanomaterials of larger sizes were formed started. Therefore, method 1 was chosen to carry out this comparative study of SiO_2_-NPs synthesis.

In recent years, many studies have reported the production of NPs by heating *via* MW, for all cases, in addition to the acceleration of the process and energy savings, there were more homogeneous nucleation and better growth conditions for NPs, providing evidence that microwave synthesis is a promising process.^38–41^ The time and power parameters, programmed for the synthesis processes employing MW irradiation, are those of most significant importance in various studies for the application of this heating method.^26–28,42–44^ The behavior of the system as a whole, is dependent on thermal and non-thermal effects that may arise from interactions of the reaction medium with microwaves leading to the modifications that are responsible for the formation of SiO_2_-NPs.^26–28,44^ As reported by Peres *et al.* (2018)^44^ chemical bonds are not formed or destroyed for the formation of these NPs from the application of MW, which justifies the formation of materials with characteristics similar to those produced in a conventional way. Thus, the DLS and *ξ*-potential of SiO_2_-NPs were analyzed and compared for both synthesis processes.

**Fig. 1:**
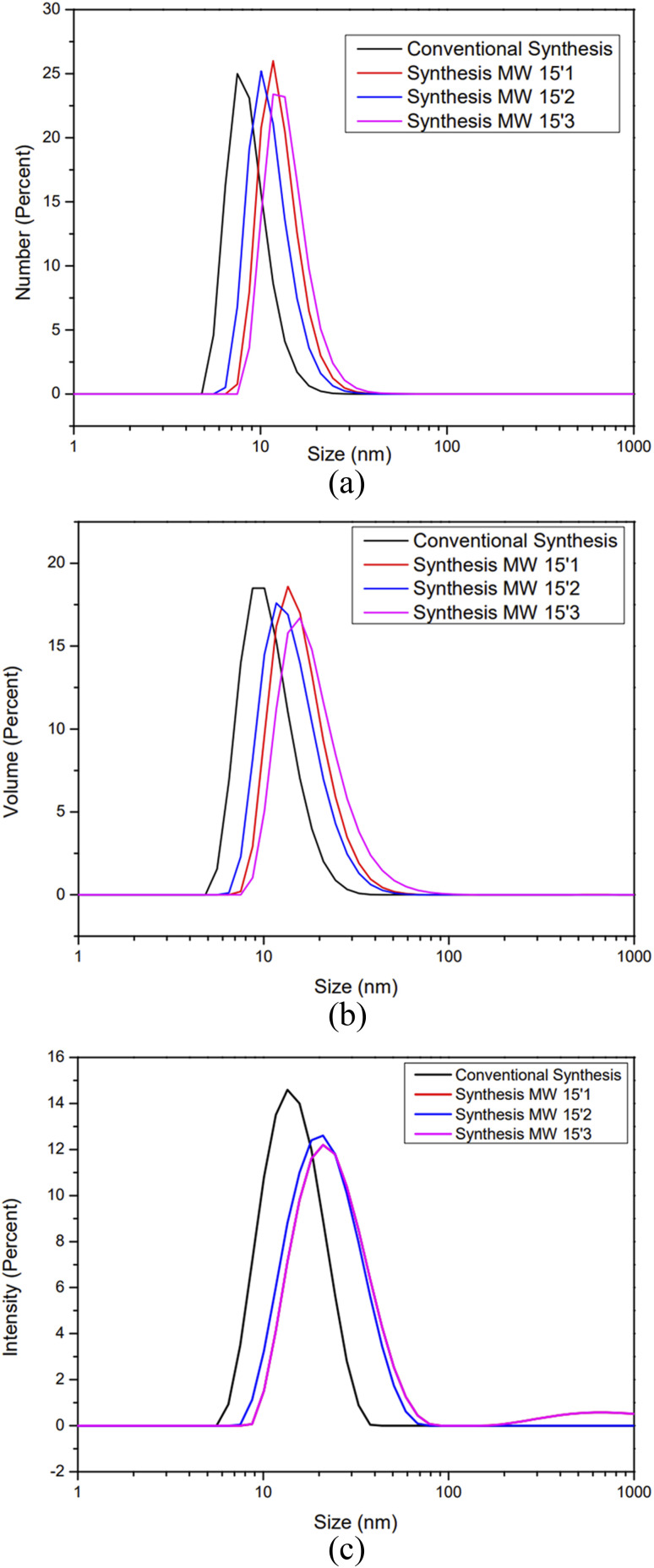
(a) DLS number of silica nanoparticles, through the comparison of conventional and microwave synthesis (in triplicate). (b) DLS volume of silica nanoparticles through the comparison of conventional and microwave synthesis (in triplicate). (c) DLS particle size distribution of silica nanoparticles through the comparison of conventional and microwave synthesis (in triplicate).

For comparative purposes with the synthesis presented in this work, it would be necessary that the characterization techniques were similar, so the similar technique between the synthesis and adopted as a comparative parameter is the transmission electron microscopy. The DLS results (Fig. 1a–c) were obtained from program 1 (Table 1). The responses exhibit single narrow Gaussian distribution for all replicates, indicating adequate robustness of the proposed method, therefore, it was the program chosen for comparison. The DLS results (11.57 ± 0.37 nm) described in Table 3 attested that program 1 was closest in terms of particle size compared to the conductive synthesis (8.3 ± 0.26 nm). Additionally, all of the PdI results obtained with the program 1 were smaller than 0.300, indicating interesting results regarding the dispersion of the nanoparticles. The stability of the prepared SiO_2_-NPs was also investigated using the *ξ*-potential, and the results are shown in Table 4.

**Table 3:** DLS results obtained for SiO_2_-NPs produced from microwave heating programs in Table 1^a^

Microwave heating (program)	Size	*F*_calc_	*t*_calc_
1	11.57 ± 0.37	2.00	12.3
2	12.83 ± 1.04	15.50	7.3
3	16.58 ± 2.44	85.04	—
4	21.61 ± 6.85	670.30	—
Conductive heating^b^	8.30 ± 0.26	—	—

a Tabulated values of *n* = 3 and *p* = 0.05.

b Values obtained from ref. 24.

**Table 4:** Zeta potential results obtained for SiO_2_-NPs produced from conductive and microwave heating for synthesis

Microwave heating (program)	*ξ*-Potential (mV)
1	−31.8
2	−30.8
3	−31.9
4	−33.4
Conductive heating	−30.8

From the results using program 1 (see Table 1), adequate stability of the produced SiO_2_-NPs was attained, attesting the zeta potential values^45–47^ as −31.8 mV, and closest than obtained through conductive heating (−30.8 mV), and for heating *via* MW we did not find values to compare with methods 2, 3, and 4 (Table 1). The results presented allow us to affirm that the synthesis performed *via* MW presented satisfactory results in a shorter time interval, with better stability of the synthesized material and improvement in the robustness of the synthesis method with heating *via* MW, allowing homogeneous materials in size, dispersion, and stability.

**Fig. 2:**
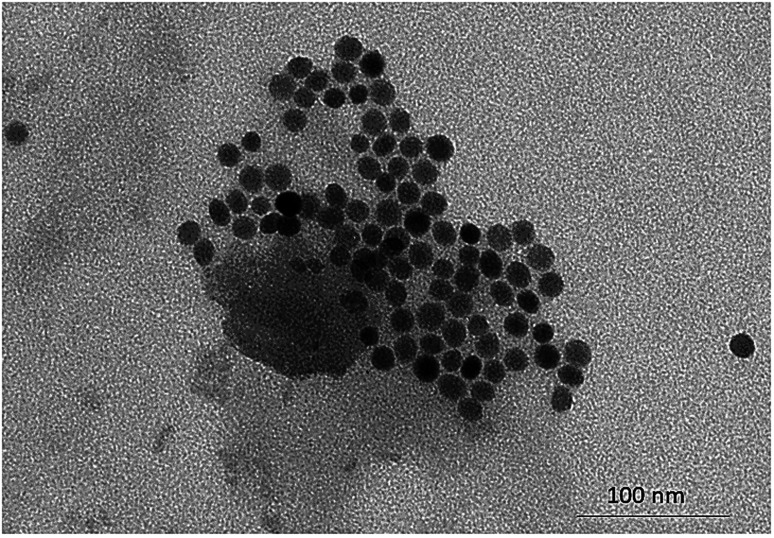
TEM micrographs of silica nanoparticles MW synthesis for evaluating morphology.

**Fig. 3:**
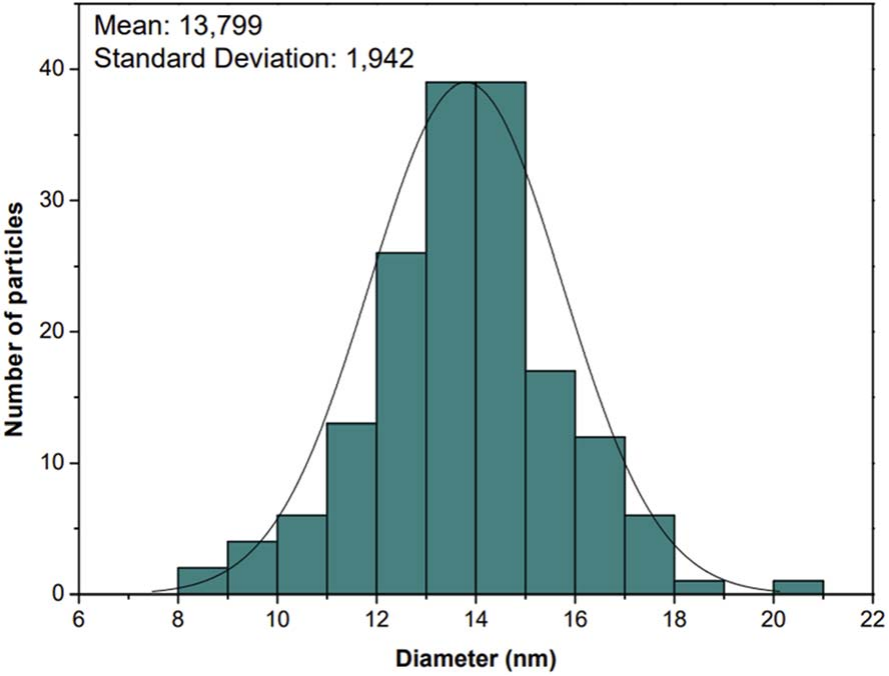
Particle size histogram of the silica nanoparticles distributions MW synthesis.

According to the literature, stable syntheses for small-sized (*ca.* 10–20 nm) SiO_2_-NPs from sodium silicate solution are rare,^22,25,42,48,49^ and reports were found in the literature about the importance of MW power and time applied in the synthesis of NPs of different natures.^26–28,42–44^ This work observed that the shorter the time at higher power at the synthesis level (700 W for 15 min), the more homogeneous the DLS results obtained. On the other hand, it was observed that standing time above 25 min leads to NP cluster formation (Fig. SM2 – ESI†); this can be seen in the widening of the distribution and the significant increase in PdI. The TEM images (Fig. 2) show the spheroidal morphology of the SiO_2_-NPs synthesized by MW, and the particle size distribution (Fig. 3) indicates the average NP size of 13.80 ± 1.94 nm, which is corroborated by DLS results (11.57 ± 0.37 nm) and is statistically similar (*p* < 0.05; *n* = 3).

The results obtained by microscopy and DLS (Fig. 1–3) are directly associated with the heating method employed, since particles containing effective or partial charges when interacting with microwaves through the phenomena of dipole rotation and ion migration, are responsible for the rapid and homogeneous heating of the medium in periods sufficiently adequate to achieve satisfactory results in terms of size distribution, surface area, volume and diameter of SiO_2_-NPs.^42,44^ The power that is directly related to the number of microwaves irradiated, in this case, was a preponderant factor for the occurrence of non-thermal phenomena, since the application of method 1 confirmed that the shorter the exposure time of the system using the highest power, the better these results. Another important characteristic of these NPs was the results obtained from TEM responsible for indicating the crystallinity of SiO_2_-NPs, the results show high purity of the material, a factor that may also be influenced by the heating method employed, since residual materials may have been degraded or volatilized in conventional methods. The microwave route provided material with better characteristics for application in transgenic soybean crops, with higher values of stability, particle volume, particle diameter, and intensity in size. In addition, microwave synthesis provided material with higher purity in a shorter time (30 min). This was because, compared to traditional external heating methods, microwave heating is rapid volumetric heating without the process of heat conduction, which can achieve uniform heating in a short period of time, and as a consequence, the production of NPs is facilitated, similar to other materials synthesized *via* MW heating.^42,44^

Other works reported in the literature indicate the use of MW technology in the synthesis of NPs due to the improvement in the production of these materials, as well as the economy that the technology makes possible. Corradi *et al.* (2006)^42^ synthesized colloidal spherical nanoparticles of monodisperse silica from the hydrolysis and condensation of tetraethylorthosilicate (TEOS) using a continuous microwave synthesis process. The authors optimized the condition required to obtain silica nanopowders and not agglomerate nanoparticles were used for this purpose, and the results were compared with those obtained in batch systems. In the results performed for this synthesis, the average particle diameter became less than 50 nm as the residence time was decreased.^42^ Peres *et al.* (2018)^45^ synthesized SiO_2_-NPs *via* MW technology from rice husk for remediation of azo dyes in aquatic systems. For comparative purposes, the authors compared the material synthesized by MW with SiO_2_ nanopowder standards, and through this, it was possible to prove that heating *via* MW enabled the production of SiO_2_-NPs with high purity, better size distribution, porosity, morphology as well as pore diameter and pore volume. Similarly, other NPs in which MW technology was used for the NPs formation process obtained satisfactory results,^26,27^ in the green synthesis of SeNPs, as well as in SmCo_5_ magnetic particles and in both materials improvements were observed in the syntheses and their characterizations. These facts and the results of this work corroborate that MW technology for the development of a method for the synthesis of SiO_2_-NPs are promising, demonstrate improvements in the homogeneity of the materials, and that in addition to being environmentally friendly are economically viable.

The results of size distribution, stability, and morphology of the SiO_2_-NPs indicated that program 1 was the most suitable for synthesis. For the comparative study, statistical tests (*F* and *t*) were performed using the results of the syntheses carried out by microwave heating and the conductive heating method, which consisted of a conductive heating system with reflux and 24 h of synthesis. Table 3 shows the results of the *F* and *t*-tests for *p* < 0.05 (*n* = 3). According to the tests, the DLS results obtained for programs 1 and 2 (see Table 1) indicate no significant difference in terms of size distribution of the SiO_2_-NPs. However, all SiO_2_-NPs obtained from the microwave synthesis differ in size from those obtained *via* conductive heating synthesis at a 95% confidence interval.

**Fig. 4:**
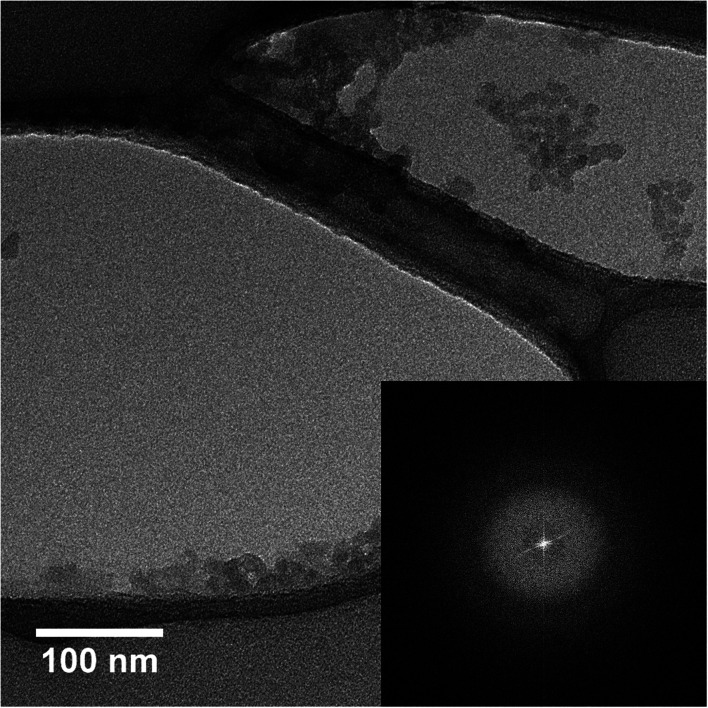
TEM micrograph of silica nanoparticles MW synthesis for evaluating crystallinity.

From the results obtained from all the techniques discussed in this study, it is possible to correlate all of the responses obtained between such techniques. For example, DLS and TEM results (from program 1, Table 1) state that these responses are correlated in size distribution and average size, as well as homogeneity in the formation of NPs. In fact, the exposed results provide evidence that nanoparticles are stable (*ξ*-potential: −31.80 mV) and have small sizes. Regarding the crystallinity of the presented SiO_2_-NPs, it can be observed that they are amorphous, which was confirmed with the micrograph presented *via* TEM with fast Fourier transform (see Fig. 4), since amorphous silica is usually obtained when used temperatures below 800 °C.^45,50^ As for this method there was no temperature control during the synthesis, it is possible to estimate them through the applied power and the synthesis time of the NPs, with this, during the synthesis it is possible to reach temperatures higher than 300°C, as well as to reach higher pressures. For heating *via* MW, Corradi *et al.* (2006)^42^ demonstrate by micrographs that SiO_2_-NPs are spherical and homogeneous and have an average value smaller than 50 nm. Peres *et al.* (2018)^45^ obtained synthesis of SiO_2_-NPs by heating *via* MW from rice husk, and from the surface images (SEM) the authors found an average size of 93 nm, values close (*ca.* 90 nm) to the MW synthesis of Ag@SiO_2_ core–shell NPs proposed by Daublytė *et al.* (2021),^28^ these reported values are much higher than the average size of the NPs of the method that are present in this work.

From these optimizations, a uniform distribution of NPs was obtained using program 1 (Fig. 2 and 3, and Table 1). However, on an industrial scale, the continuous manufacturing and collection processes can give access to mass-scale production of inorganic materials with relatively small facility, compared to the conventional processing method.

Finally, the total Si quantification (1.05% ± 0.02%) was obtained through an external calibration *via* ICP OES. For checking the accuracy, the Si determination in the certified reference material (CRM 1640a) was 5.177 ± 0.101 mg kg^−1^, which agreed with the certified value of 5.169 ± 0.017 mg kg^−1^.

### 3.2 Applications of SiO_2_-NPs

The silica nanoparticles synthesized by microwave heating were applied to the SiO_2_-NP-contaminated soil of soybean crops as a control soil culture to evaluate the behavior of these crops under the influence of stresses caused by the NPs. In fact, NPs having a diameter of 5–20 nm, as the nanoparticles obtained through this work, might penetrate through the cell wall and easily reach the plasma membrane.^51^ These nanoparticles can pass through stomata openings or the base of trichomes, then foliar spray them into different tissues. Then, some changes in different cellular and physiological functions of the plant are attained after the accumulation and translocation of NPs.^51^

One of these changes refers the nanoparticles can alleviate metals toxicity such as Zn, Cu, Cd, Fe, Al, and Mg.^51^ It is clear that the mechanism is dependent on the plant variety. In soybean, this is the first time that SiO_2_-NPs are being applied, but species such as sodium silicates and alkaline silica may affect the rhizosphere pH, resulting in a decrease of metal concentration in the soil,^52^ and consequently lower metal concentration in the upper parts of the plants. The Cu accumulation in the leaves can be observed through the Si accumulation in the endodermis, a similar result as the behavior of heavy metal-treated plants in terms of antioxidant enzyme control.^53^ On the other hand, the photosynthetic rates are controlled and sustained by silica nanoparticles, once the chloroplasts are stabilized, maintaining the integrity of the photosystem, and increasing the content of pigments. SiO_2_-NPs were also effective in alleviating the Fe excess toxicity in rice^54^ and enhancing the oxidative power of Fe from ferrous ions in rice roots. Also, in the Zn and Cd polluted soil, Zn was found to coexist with silica in the form of a Zn–silicate complex,^51^ in a manner similar to that observed in Zn uptake in both transgenic soybean leaves that were treated with SiO_2_-NPs in this work.

The total content of Cu, Ni, and Zn in leaves was evaluated (see Table 5), and the results for Zn and Ni demonstrate a statistical difference (at 95% confidence interval) between RR and Intacta soybeans, which were grown in the same conditions, and the control and SiO_2_-NPs groups.

**Table 5:** Determination of Ni, Zn and Cu (mg kg^−1^) in transgenic soybean leaves by ICP-MS (*n* = 3)^a^

*m*/*z*	Control	SiO_2_-NPs
**Roundup ready – RR**		
^60^Ni	5.14 ± 0.28	4.36 ± 0.03
^65^Cu	11.00 ± 0.64	10.79 ± 0.80
^66^Zn	51.73 ± 1.81	44.65 ± 2.45

**Intacta**		
^60^Ni	9.14 ± 1.81	4.71 ± 0.48
^65^Cu	11.84 ± 0.38	7.56 ± 0.07
^66^Zn	44.52 ± 2.13	24.90 ± 1.77

a All statistical tests were applied ANOVA for *p* = 0.05 and *n* = 3.

For Cu, the results between the same transgenic crops and different growing conditions showed no significant difference, which did not affect the uptake of this metal in these crops, as well as between the control groups of both transgenes. However, the only comparison that shows a difference in the concentration of Cu was between the cultures containing SiO_2_-NPs in different types of transgenes. Therefore, it can be inferred that for soybean cultivated in soils containing SiO_2_-NPs there are different behaviors towards the same emerging contaminant (as nanoparticles).

**Fig. 5:**
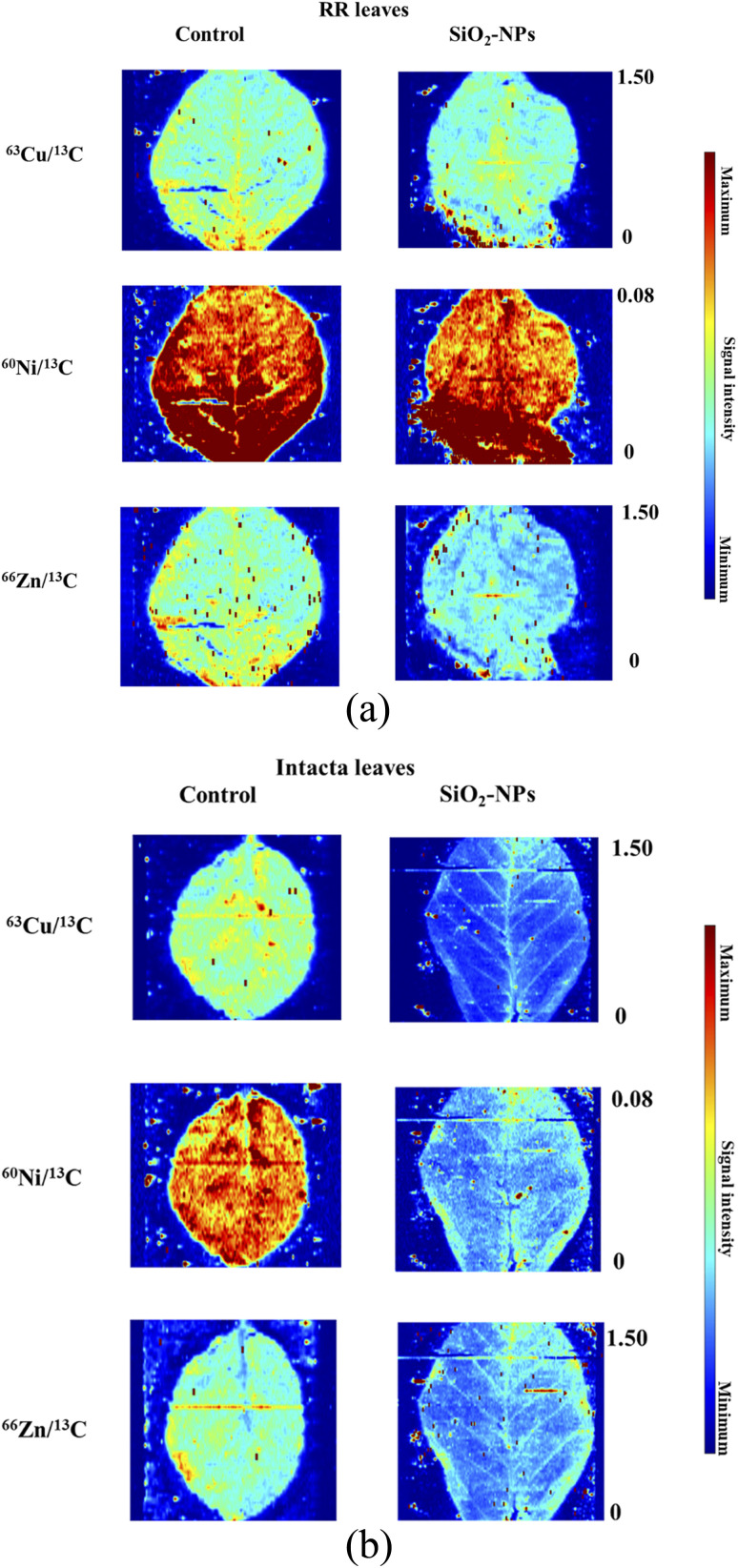
(a) Elemental spatial distribution in RR leaves sample with and without SiO_2_-NPs contamination. (b) Elemental spatial distribution in Intacta leaves sample with and without SiO_2_-NPs contamination.

In order to deeply evaluate such results, the LA-ICP-MS imaging^9,34^ of micronutrients as Cu, Ni, and Zn were then performed at micrometer resolution. After observing the images, the spatial distribution of metals on the surface of the RR and Intacta soybean leaves was verified, as was the signal intensity by the control and the NPs' influence. In the RR leaf samples (Fig. 5a), similarities between the elemental distribution and the signal intensity on the sample surface were observed, in which the control sample shows Cu, Ni, and Zn accumulation near the leaf stem.

In the Intacta leaf samples (Fig. 5b), the impact of the SiO_2_-NP crop contamination is clearly observed because there is the highest signal intensity of Cu, Ni, and Zn on all sample surfaces.^9,34^

Then, it can be qualitatively observed that the leaves of the group with the SiO_2_-NPs did undergo drastic changes, corroborating with previous studies employing SiO_2_-NPs.^9,24^

The presence of NPs in the environment can affect the homeostasis mechanism of plants, and changes may occur in the absorption of macro- and/or micronutrients in the different plant compartments.^55,56^ Thus, the differences found for Cu, Ni, and Zn, both in the total quantification of leaves *via* ICP-MS and also in the intensities obtained after ablation *via* LA-ICP-MS, may be associated with the stress condition of both transgenic crops compared to their control groups. Similarly, some differences were observed in the literature regarding the formation of reactive oxygen enzymes (ROS), carbonic anhydrase, superoxide dismutase, and antioxidant enzymes, which are associated with high absorption of Cu and Zn.^17,19,57^ Considering Ni, a difference was observed (except between the control groups of both transgenes), which may be associated with the natural defense mechanisms of plants through the use of biomolecules regulating their cellular balance. For example, the ZIP, NRAMP, Yellow Stripe (YS), and Copper Transporter (COPT) families play a primary role in the uptake and remobilization of metals from intracellular compartments to the cytosol. Such an effect was observed in *Arabidopsis thaliana*,^58^ where the ZIP family, comprised of 15 genes involved in the transport of cations (including Zn, Mn, Fe, Ni, and Cd) across cell membranes into the cytoplasm, contributes to metal homeostasis.^56,57^

The images of the distribution of micronutrients reaffirm that plants use metal transporters in controlling the homeostasis of metal cations in their natural process of maintaining the balance of nutrients in their compartments due to the high concentration of SiO_2_-NPs. This fact highlights that metal transporters play a key role in the transport, distribution, and compartmentalization of ions in plant tissues and cells. Thus, some reports in the literature have identified different families of transporter genes functioning in the plant cell membrane that assist in the uptake, translocation, intracellular transport, and detoxification of transition metals. Among them are heavy metal ATPases (like CPx), natural resistance-associated macrophage proteins (NRAMPs), cation diffusion facilitators (CDFs), the ZIP family, and anti-cation carriers.^57,59,60^

In fact, we can see that the elements evaluated play fundamental roles in essential biological processes, in which they act as regular elements and cofactors of metalloproteins involved in plant systems, such as respiration, photosynthesis, or protection against oxidative stress.^57,59,60^

## 4. Conclusions

In this study, a new methodology employing MW heating was established for the synthesis of SiO_2_-NPs, presenting some advantages over conductive synthesis. In fact, the results obtained provided evidence that NPs are stable, show reduced size, and are cost-effective. In fact, the methodology is 30 times faster than conductive heating with lower operating costs (US$ 0.50 per synthesis) when compared to synthesis *via* conductive reflux heating (US$ 9.0 per synthesis). Additionally, for this new synthesis method, in addition to energy and operational gain, it can be considered environmentally friendly, as the volume of reagents used was 4 times smaller.

Additionally, after the microwave-based SiO_2_-NP synthesis, such NPs were then applied to a soybean culture, and it was clear that through our ICP-MS results and corroborated thorough bioimaging employing LA-ICP-MS, the absorption of some micronutrients (Cu, Ni, and Zn) was affected due to the presence of SiO_2_-NPs. In fact, due to the advantages of MW-based synthesis over conventional ones, becomes attractive new studies focusing on these NPs applications as nanofertilizers or as contamination mitigating agent.

## Author contributions

Daniel C. Carneiro: conceptualization, experimental, data analysis, wrote the original manuscript and designed the figures. Danielle S. Francischini: experimental, data analysis for laser ablation. Italo O. Mazali: experimental, data analysis TEM evaluation, help in writing the original manuscript. Fernando A. Sigoli: experimental, data analysis TEM evaluation, help in writing the original manuscript. Marco A. Z. Arruda: conceptualization, coordinator and advisor in this work and wrote the original manuscript.

## Conflicts of interest

The authors declare no potential conflict of interest.

## Supplementary Material

RA-013-D3RA05648A-s001
