# Supplementary material for: The microwave-assisted synthesis of silica nanoparticles and their applications in a soy plant culture
Source: RSC Adv. 2023 Sep 18;13(39):27648–56. doi: 10.1039/d3ra05648a (PMC10505942; doi:10.1039/d3ra05648a)
Supplement: RA-013-D3RA05648A-s001 [file RA-013-D3RA05648A-s001.pdf]

Electronic Supplementary Material (ESI) for RSC Advances.
This journal is © The Royal Society of Chemistry 2023

# Microwave-assisted synthesis of silica nanoparticles and their applications in a soy plant culture

Daniel Carneiro Freitas, Italo Odone Mazali, Fernando Aparecido Sigoli, Danielle da Silva Francischini and Marco Aurélio Zezzi Arruda

## Supplementary material

### FIGURE SM1

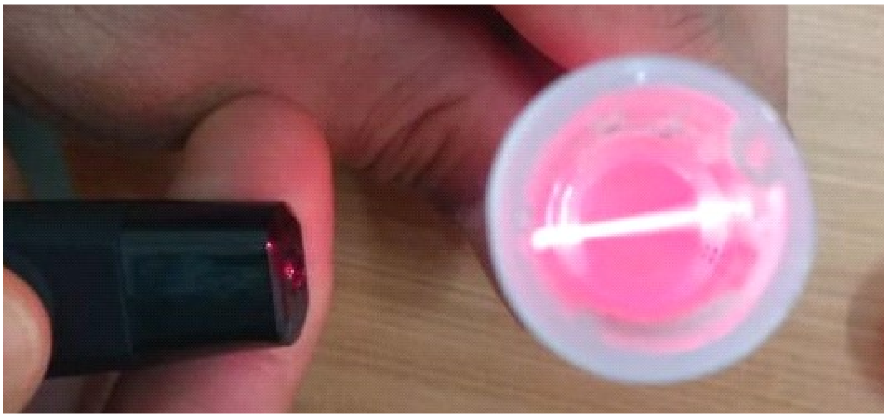

The Tyndall effect in the sample after the synthesis of $SiO_2$-NPs by microwave

**Figure SM2**

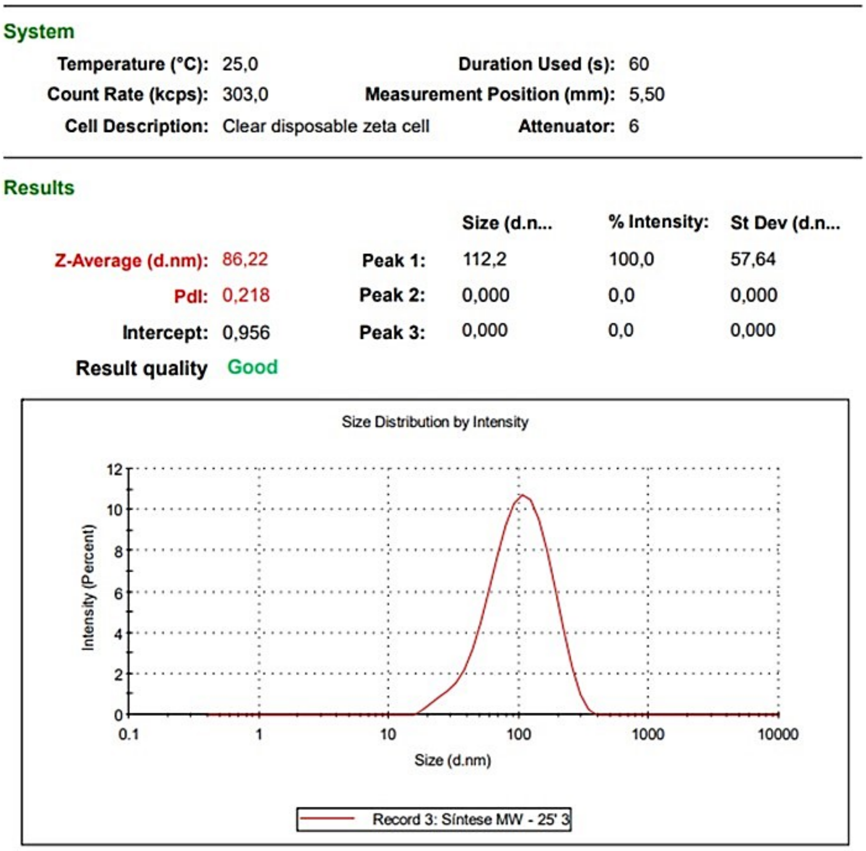


DLS analysis showing clusters formation with microwave time of 25 min. in step 2. (see Table 1)
